# The HTLV-1 Tax protein binding domain of cyclin-dependent kinase 4 (CDK4) includes the regulatory PSTAIRE helix

**DOI:** 10.1186/1742-4690-2-54

**Published:** 2005-09-15

**Authors:** Kirsten Fraedrich, Birthe Müller, Ralph Grassmann

**Affiliations:** 1 Institut für Klinische und Molekulare Virologie, Universität Erlangen-Nürnberg, Schlossgarten 4, D-91054 Erlangen, Germany

## Abstract

**Background:**

The Tax oncoprotein of human T-cell leukemia virus type 1 (HTLV-1) is leukemogenic in transgenic mice and induces permanent T-cell growth *in vitro*. It is found in active CDK holoenzyme complexes from adult T-cell leukemia-derived cultures and stimulates the G1- to-S phase transition by activating the cyclin-dependent kinase (CDK) CDK4. The Tax protein directly and specifically interacts with CDK4 and cyclin D2 and binding is required for enhanced CDK4 kinase activity. The protein-protein contact between Tax and the components of the cyclin D/CDK complexes increases the association of CDK4 and its positive regulatory subunit cyclin D and renders the complex resistant to p21^CIP ^inhibition. Tax mutants affecting the N-terminus cannot bind cyclin D and CDK4.

**Results:**

To analyze, whether the N-terminus of Tax is capable of CDK4-binding, *in vitro *binding -, pull down -, and mammalian two-hybrid analyses were performed. These experiments revealed that a segment of 40 amino acids is sufficient to interact with CDK4 and cyclin D2. To define a Tax-binding domain and analyze how Tax influences the kinase activity, a series of CDK4 deletion mutants was tested. Different assays revealed two regions which upon deletion consistently result in reduced binding activity. These were isolated and subjected to mammalian two-hybrid analysis to test their potential to interact with the Tax N-terminus. These experiments concurrently revealed binding at the N- and C-terminus of CDK4. The N-terminal segment contains the PSTAIRE helix, which is known to control the access of substrate to the active cleft of CDK4 and thus the kinase activity.

**Conclusion:**

Since the N- and C-terminus of CDK4 are neighboring in the predicted three-dimensional protein structure, it is conceivable that they comprise a single binding domain, which interacts with the Tax N-terminus.

## Background

The Tax protein of human T-cell leukemia virus type 1 (HTLV-1) is an essential regulator of viral replication and a critical determinant of the HTLV-induced diseases. These include the aggressive and fatal malignancy of CD4^+ ^T-lymphocytes termed adult T-cell leukemia (ATL) [1-3]. Several lines of evidence indicate that p40^*tax *^is the oncogene responsible for viral lymphocyte-transforming and leukemogenic properties [4-7]. Mechanistically, several biochemical features of the protein can cooperate to transform, among them transcriptional stimulation of cellular signal transducers, cytokines [8-11] and anti-apoptotic effectors. Tax' capacity to stimulate aneuploidy and to interfere with DNA repair [12] could indirectly support malignant progression. A major mechanistic explanation for the mitogenic and immortalizing effects of the Tax oncoprotein is provided by its ability to stimulate the G1- to S-phase transition in T-cells [6,13-15].

In mammalian cells, G1-progression is controlled by the sequential activation of several cyclin-dependent kinases (CDKs), starting with CDK4, CDK6 and CDK2. Tax activates CDK4, CDK6 and CDK2 leading to phosphorylation of retinoblastoma (Rb) tumor suppressor proteins and liberation of the transcription factor E2F [6,16]. Moreover, Tax may also induce Rb degradation [17] and increases cellular E2F synthesis [18,19]. Several indirect effects of Tax and features of HTLV-infected cells may support the impact of Tax on CDK. For example, HTLV-1-infected T-cells contain increased levels of cyclin D2 [16,20,21], which upon binding to CDK4 forms functional holoenzyme complexes. Cyclin D2 expression is upregulated by interleukin-2 receptor (IL2-R) signals [22-24]. Tax may cooperate with interleukin-2 (IL-2) signaling either indirectly through stimulating the expression of IL-2Rα or directly by activating the cyclin D2 promoter [21,25]. Furthermore, expression of CDK inhibitory proteins, like p18^INK4C ^[20], p19^INK4D ^and p27^Kip1^[16,26] is reduced in the presence of Tax. By contrast, the inhibitory protein p21^CIP1 ^is strongly upregulated in Tax-containing cells [20,27]. Tax also represses the function of distinct tumor suppressor proteins which interfere with G1- to S-phase transition. These include p16^INK4A^, p15^INK4B ^[26,28,29] and p53 [30-35].

The protein-protein contact with the components of the cyclin D/CDK complexes provides a major explanation for the G1-phase stimulating effects of Tax. The Tax interaction with the CDK and cyclin component is direct and specific. This interaction is detectable *in vitro*, in transfected fibroblasts, HTLV-1-infected T-cells, and ATL-derived cultures [36,37]. The Tax-CDK complex represents an active holoenzyme. Direct association with Tax enhances CDK4 activity. This increased kinase activity in the presence of Tax may be explained by intensified association of CDK4 and its positive cyclin regulatory subunit and by resistance of the complex to inhibition by p21^CIP1 ^[36,37].

To understand the molecular mechanism of the Tax-mediated CDK4 activation, the interacting domains of Tax and CDK4 were characterized. Here we show that a segment of 40 amino acids derived from the N-terminus of Tax is sufficient to bind CDK4 and cyclin D2. To define a Tax-binding domain, a series of CDK4 deletion mutants was tested in different assays. These point at two regions derived from the N- and C-terminus of CDK4 which upon deletion consistently result in reduced binding capacity. The potential of these isolated regions to interact with Tax was demonstrated by mammalian two-hybrid analysis. These experiments concurrently revealed Tax-binding at the N- and C-terminus of CDK4.

## Results and discussion

### Capacity of the isolated N-terminus of Tax to bind cyclin D2- and CDK4

**Figure 1:**
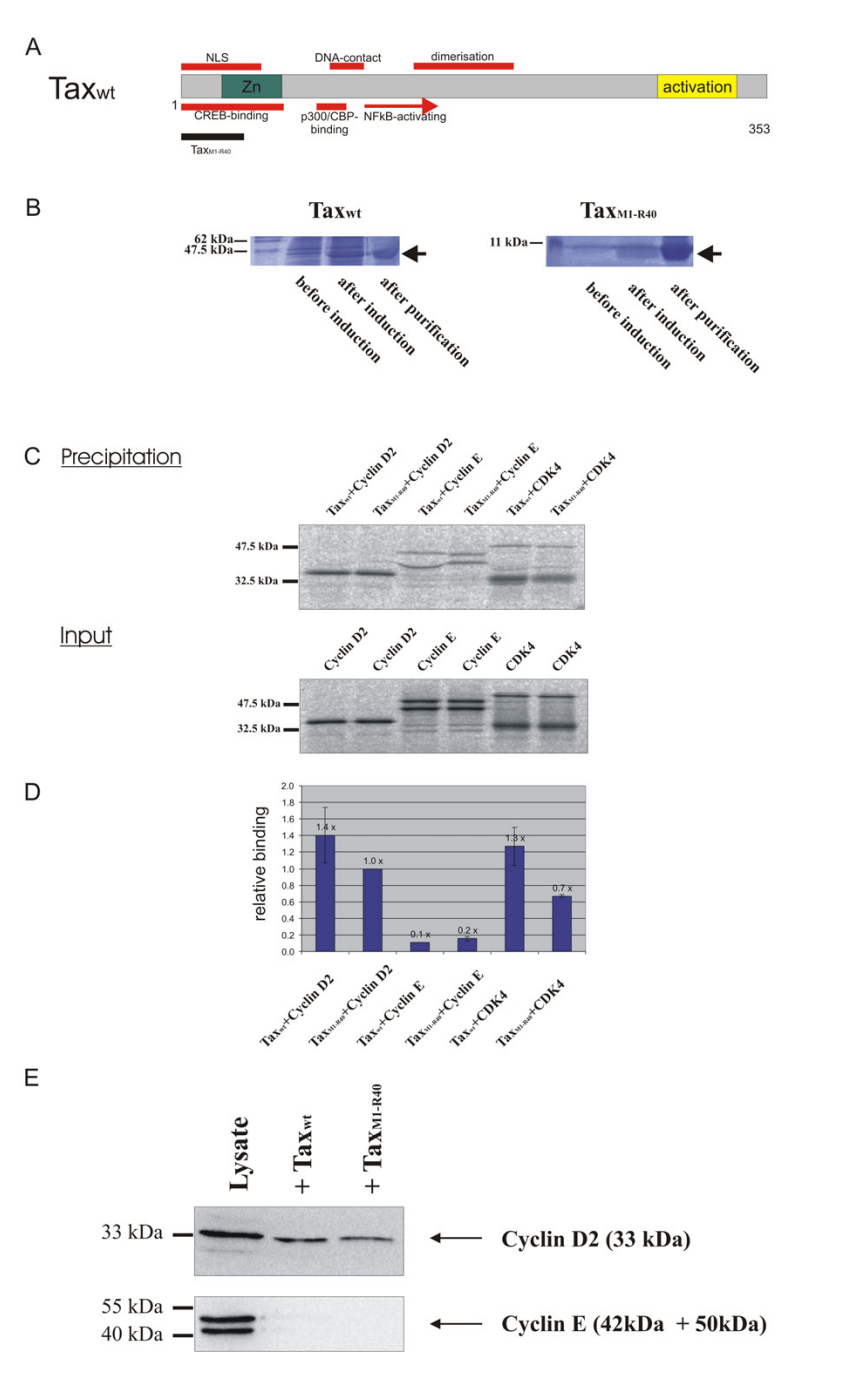
**Binding of the isolated Tax N-terminus to CDK4 and cyclin D2. **A) Physical map of Tax's functional domains and the position of the N-terminal peptide B) Tax_wt _and Tax_M1-R40 _were produced in *E. coli *and coupled to S-protein agarose. The figure depicts a coomassie brilliant blue-stained SDS-PAA gel loaded with the purified protein coupled to S-protein agarose and samples before and after induction with IPTG. C) CDK4, cyclin D2 and cyclin E were translated *in vitro *and incubated with S-agarose coupled, *E.coli*-produced Tax_wt _and TaxM1-R40. Bound proteins were detected in gels by phosphoimaging (precipitation). To control for equal inset, aliquots of the radioactive proteins were subjected to gel electrophoresis (input). D) The radioactive signals of bound proteins of two independent experiments were quantitatively evaluated. The figure depicts the mean relative binding. E) For *in vivo *pull-down analysis, cyclin D2 and cyclin E plasmids were transfected into 293T cells. Lysates were incubated with S-agarose coupled to Tax_wt _or the N-terminal peptide (Tax_M1-R40_). Bound proteins and aliquots of the lysates were subjected to gel electrophoresis and immunoblotting, using polyclonal cyclin D2 and cyclin E antibodies.

N-terminal Tax mutants bind neither CDK4 nor cyclin D2 and are incapable to stimulate CDK holoenzyme activity. This indicates that the region is required for binding and activation. To investigate whether this segment is also sufficient for binding to cyclin D2 and CDK4, the coding sequence of the N-terminal fragment (codons 1–40) was cloned into the prokaryotic expression vector pET29b+ (Figure 1A). The corresponding protein (Tax_M1-R40_) and Tax_wt _were produced in *E. coli *and coupled to S-protein agarose (Figure 1B). To demonstrate direct interaction, *in vitro *binding assays were performed. For this purpose, ^35^S-labeled cyclin D2, CDK4 and, as a control, cyclin E were synthesized *in vitro*. All *in vitro *translation reactions resulted in major bands of the expected size in equal amounts (Figure 1C Input). Cyclin E was produced in two previously observed isoforms [38]. Bands of minor intensity are most probably due to incorrect in vitro translation products and were ignored for quantitation. For binding analysis aliquots of the agarose-coupled Tax_M1-R40 _and Tax_wt _(Figure 1B) were incubated with the *in vitro*-translated proteins. As Figure 1C (Precipitation) shows, incubation with Tax_M1-R40 _and Tax_wt _resulted in significant amounts of cyclin D2 and CDK4. By contrast, both of the cyclin E isoforms were significantly less precipitated. Three independent experiments were quantitated. They revealed a 3.5 – 5 fold increased protein binding of Tax_M1-R40 _to CDK4 and cyclin D2 compared to the cyclin E control (Figure 1D). The binding to CDK4 of the N-terminal peptide compared with full length Tax was slightly reduced. This may indicate structure differences rather than the contribution of other Tax regions in CDK4 binding. The interaction of the N-terminal Tax fragment with cyclin D2 could be reproduced with natural folded proteins in pull down experiments (Figure 1E). Cyclin D2- and cyclin E-containing lysates derived from transfected 293T cells were incubated with bacterially expressed Tax_M1-R40 _and Tax_wt, _immobilized on S-agarose (Figure 1B). Subsequent analysis of bound proteins by immunoblots revealed that the N-terminal Tax peptide interacted with cyclin D2 but not with cyclin E. In summary, these results demonstrate that a N-terminal peptide of Tax, spanning amino acids 1 – 40, is sufficient for direct and specific interaction with both, cyclin D2 and CDK4. These results are in agreement with the capacity of the 40 N-terminal amino acids of Tax to bind CDK4 in a yeast two-hybrid system and in pull down analyses [39]. In extension, we demonstrated the interaction with naturally folded CDK4 protein produced in human cells. The binding of both, CDK4 and cyclin D2, by this Tax domain could cause a spacially close positioning of these proteins and thus stimulate CDK4 – cyclin D2 holoenzyme formation. This could be part of the mechanistic explanation for the enhancement of CDK4 kinase activity induced by a synthetic N-terminal Tax peptide [39]. Furthermore, this may explain the increased affinity of cyclin to CDK in the presence of Tax [36]. In addition, Tax could influence kinase activity through mediating cyclin phosphorylation by its direct contact [14]. This phosphorylation appears in cyclins which are actively complexed to cognate CDKs [40,41] and may impair cyclin degradation via the ubiquitin proteasome pathway [42].

### Relevance of N- and C-terminal CDK4 regions for Tax-binding *in vitro*

**Figure 2:**
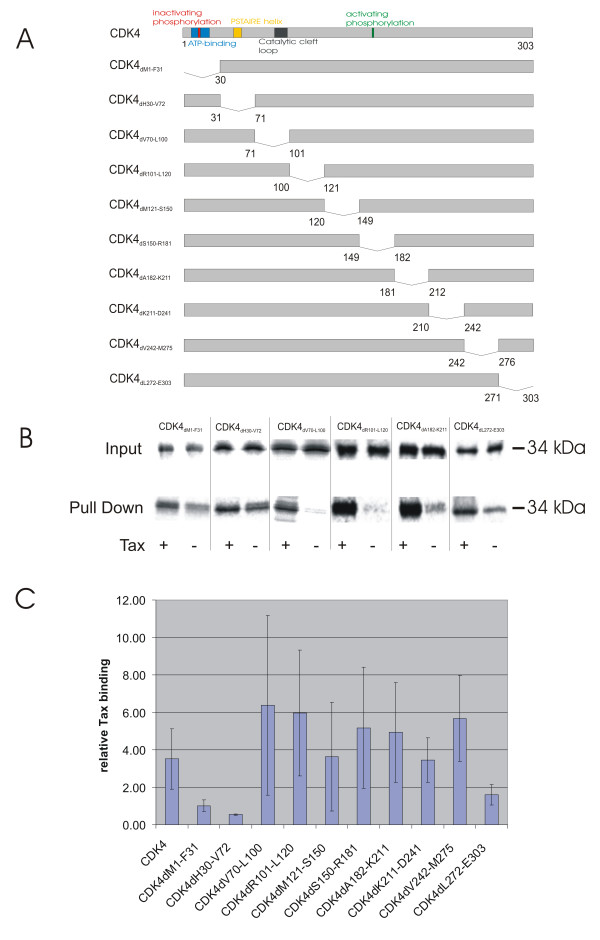
**Identification of a CDK4 region important for direct Tax interaction. **A) For binding assays, CDK4 mutants were constructed via PCR and cloned into the mammalian expression vector pcDNA3.1MycHis. B) CDK4 and its mutants were translated *in vitro *and reacted with S-agarose-coupled Tax_wt_. As a control, translated proteins were also incubated with uncoupled S-Agarose. Examples of resulting phosphorimager scans are shown. C) The diagram shows the mean Tax binding and standard deviation of three independent experiments that were quantitatively evaluated.

In order to understand whether domains, which are relevant for regulating CDK4 activity, are affected by Tax, Tax-binding CDK4 sequences were defined. For this purpose, a series of deletion mutants was generated which cover the complete coding region of CDK4 (Figure 2A). To identify CDK4 sequences, which are relevant for Tax-binding in the absence of other cellular components, *in vitro *binding assays were performed. Aliquots of the S-protein agarose matrix coupled Tax_wt _(Figure 1B) were incubated with the *in vitro*-translated, ^35^S-labeled CDK4 mutants. Subsequently, Tax-bound CDK4 mutants were collected (Figure 2B Pull down). Equal inset of the *in vitro*-translated proteins was verified (Figure 2B Input). As a background control, uncoupled S-protein agarose was incubated with the *in vitro*-translated proteins. The immobilized proteins were subjected to gel electrophoresis and quantitated by measuring the radioactivity of the specific bands. To determine relative Tax-binding, the ratio between the specific signal and the background was calculated. The results of three independent experiments (Figure 2C) show reduced relative binding compared to wild-type of three CDK4 deletion mutants in two regions. Two of them, CDK4_dM1-F31 _and CDK4_>dH30-V72_, affected a N-terminal region. In addition, a C-terminal mutant CDK_4dL272-E303 _did interact at reduced levels with Tax. Thus, the N-terminal region from amino acids 1–72 and the C-terminal region from amino acids 272–303 of the CDK4 protein directly interact with Tax. Alternatively, the deletion of these regions may reduce the protein's affinity to Tax by affecting its conformation.

### Relevance of the N-terminal CDK4 domain for binding *in vivo*

**Figure 3:**
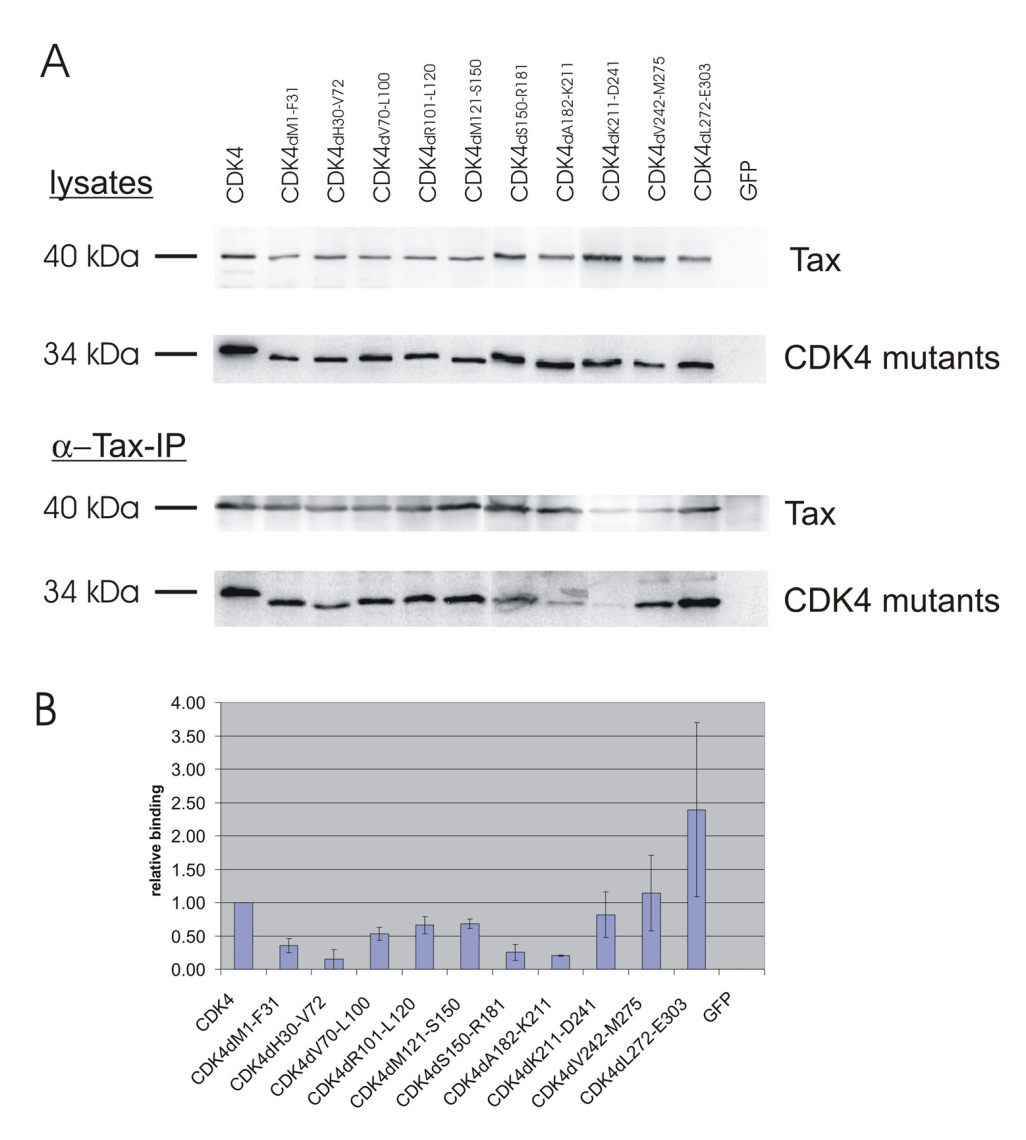
**Deletion of two regions in CDK4 interferes with Tax-binding *in vivo*. **A) Tax and CDK4 mutants were coexpressed in transfected 293T cells. The complexes were immunoprecipitated by monoclonal Tax antibodies and protein A sepharose. To detect Tax-bound CDK4 mutants, complexes and lysate controls were subjected to gel-electrophoresis and Western blotting. One representative experiment is shown. B) Luminescence emitted by specific bands of two independent experiments was quantitative evaluated and the mean relative Tax binding was calculated.

In order to characterize CDK4 sequences relevant for *in vivo *interaction, Tax and the CDK4 deletion mutants were coexpressed in transfected 293T cells in equal amounts (Figure 3A, lysates). Subsequently, coimmunoprecipitation experiments were performed (Figure 3A, α-Tax-IP) using a Tax-specific antibody. The resulting immunoblots were stained with CDK4 and Tax-specific immune reactions. These revealed a reduced affinity of Tax to some mutants, in particular to CDK4_dH30-V72 _and CDK4_dA182-K211_. To quantitate binding, the amounts of coimmunoprecipitated CDK4- and Tax-proteins were determined. The ration of both was taken as relative binding. The mean from two independent experiments shows that three CDK4 deletion mutants (CDK4_dH30-V72_, CDK4_dS150-R181_, CDK4_dA182-K211_) in two regions have significantly reduced binding affinity to Tax (Figure 3B). The mutants CDK4_dH30-V72 _and CDK4_dM1-F31_, which also appears to be reduced in binding, represent the same N-terminal region, which was identified in the *in vitro *binding assays. In addition, two mutants in the central part of CDK4 (CDK4_dS150-R181_, CDK4_dA182-K211_) resulted in reduced Tax binding. Since this central region was not required *in vitro*, its deletion may affect the CDK4 structure *in vivo*, thus rendering it inaccessible for Tax-binding. The deletion of the C-terminal amino acids (CDK4_dL272-E303_) did not affect Tax-binding, indicating that this part is not essential for *in vivo*-binding and may be replaced by cellular factors. Moreover, this result may indicate that *in vivo *the N-terminus is sufficient for Tax-binding. Thus, the *in vivo *binding experiments confirmed the relevance of the N-terminal CDK4 region for Tax-binding.

### Tax-binding activity of isolated CDK4 regions *in vivo*

**Figure 4:**
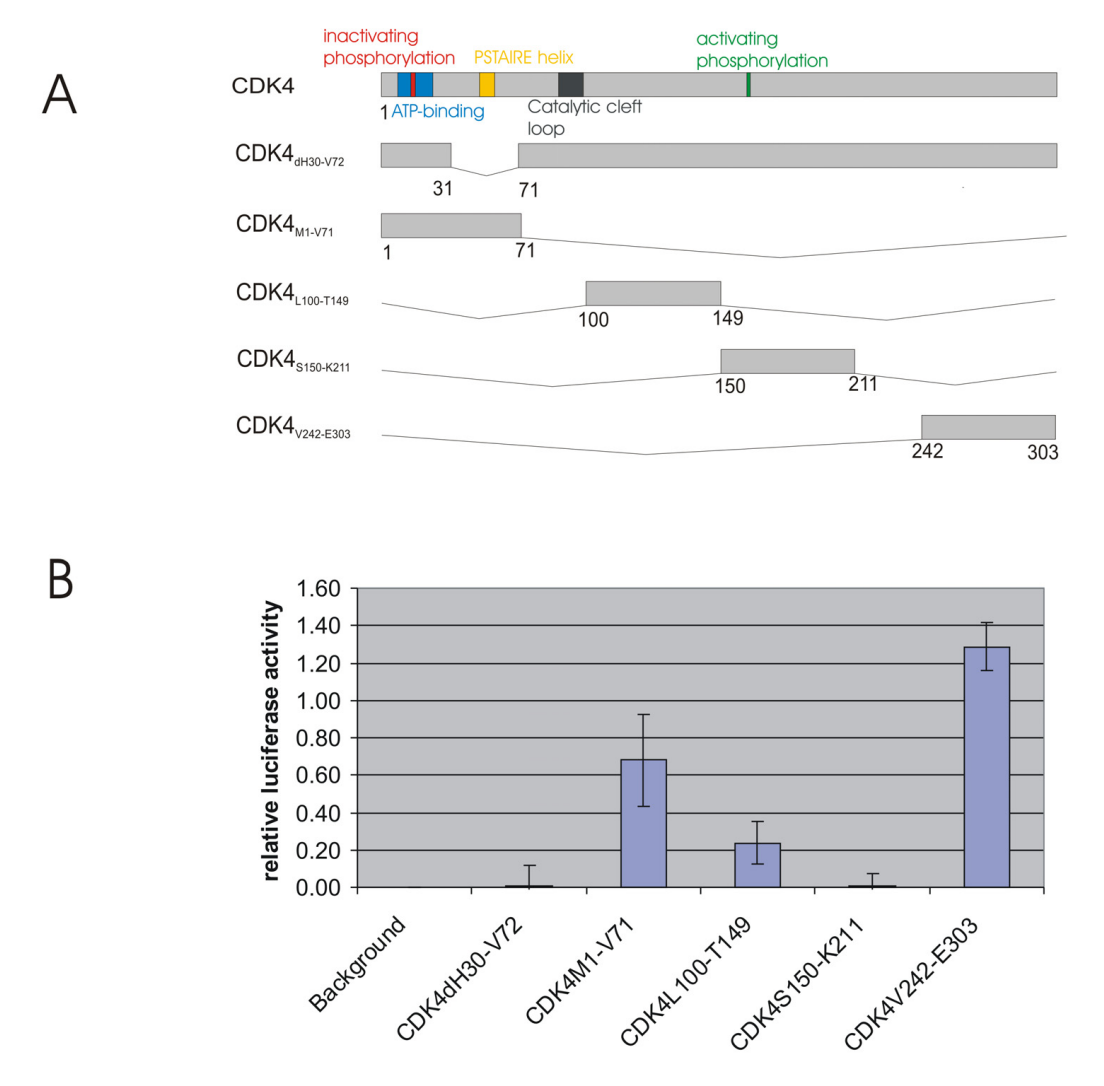
**Interaction of CDK4 and Tax peptides in an eukaryotic two-hybrid assay. **A) The coding sequence of CDK4 peptides and a CDK4 deletion mutant were constructed via PCR and assembled into the GAL4 DNA-binding domain-expressing vector. The sequence of the CDK reactive N-terminus of Tax was inserted into the VP16 activation domain-expressing vector. B) To test for interaction, CDK4-containing plasmids were co-transfected with the Tax plasmid into 293 cells and luciferase assays were performed. The mean of three independent experiments is shown.

**Figure 5:**
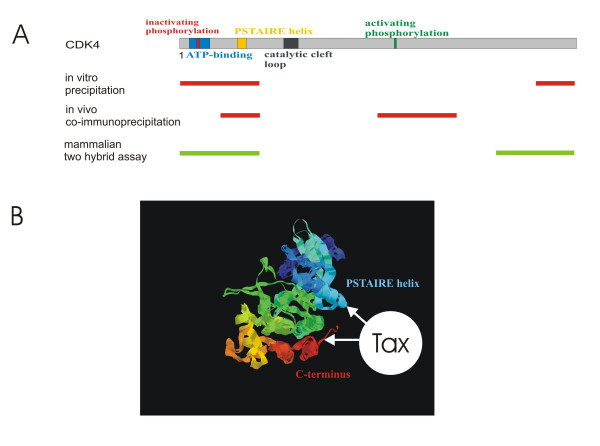
**Model of Tax-CDK4 interaction. **A) Map of CDK4 regions relevant for Tax-binding. The N-terminal region of CDK4 is relevant in all binding assays, suggesting that it is the major binding region. In addition, the C-terminus is considered as a second possible binding region. Red: regions, which upon deletion result in reduced binding; green: regions, which bind to Tax. B) Tertiary structure prediction of CDK4. The structure was calculated from the amino acid sequence at Swiss Model . The resulting pdb file was visualized with rasmol. The prediction shows the proximity of N- and C-terminal regions in the folded CDK4 protein. Thus, it is conceivable that both represent a non-contiguous binding domain for Tax. Red: C-terminal segment; blue:N-terminal segment

To investigate the affinity to Tax of those CDK4 regions, which upon deletion affected Tax-binding, mammalian two-hybrid assays were performed. All corresponding CDK-sequences were cloned into the DNA-binding domain containing vector(Figure 4A). The N-terminal region, which was found to be important for Tax-binding *in vitro *and *in vivo*, is included in plasmid pCDK4_M1-V71_. The other regions, which affected Tax-binding in only one assay, are represented by the constructs CDK4_V242-E303 _(C-terminal region) and CDK4_S150-K211 _(central region). As a control, CDK4_L100-T149 _was constructed, which contains a region whose deletion did not affect Tax-binding in all assays. In addition, the deletion mutant CDK4_dH30-V72 _was inserted into the two-hybrid vector. The coding sequence of the CDK4-binding Tax domain (amino acids M1 – R40) was assembled into the DNA activation domain containing other two-hybrid vector. To test for interaction, human fibroblasts (293 cells) were co-transfected with these constructs and luciferase assays were performed. Whereas *Firefly *luciferase indicated the binding activity, *Renilla *luciferase, which is constitutively expressed from one plasmid, was analyzed as internal transfection control. Relative luciferase activity was calculated as the ratio of *Firefly *to *Renilla *luciferase activity. The mean relative luciferase activity of three independent experiments is shown in Figure 4B. Only two of the CDK4 constructs, CDK4_M1-V71 _and CDK4_V242-E303_, yielded significant amounts of relative luciferase activity, indicating direct interaction with Tax_M1-R40_. This demonstrates that the N-terminal region of CDK4 (peptide CDK4_M1-V71_), which upon deletion reduced binding affinity *in vivo *and *in vitro*, bound Tax_M1-R40 _in the two-hybrid assay. In agreement with the notion that the binding domain is absent, the mutant CDK4_dH30-V72_, lacking 42 of these amino acids, consistently showed no binding capacity in all assays. The peptide CDK4_S150-K211_, which represents the CDK4 region affecting Tax-binding exclusively *in vivo*, revealed no binding in the two-hybrid assay. In contrast, the C-terminal peptide CDK4_V242-E303_, representing the region of CDK4 affecting Tax-binding *in vitro*, bound Tax_M1-R40_. In agreement with the other assays, the peptide CDK4_L100-T149 _did not bind. Taken together, the results of all binding assays consistently identified the CDK4 N-terminus as main interaction domain for Tax (Figure 5A). The CDK4 C-terminus, which could directly interact with Tax, may cooperate with the N-terminus, although it was not essential for Tax-binding *in vivo*.

To get an impression about the molecular interaction with the folded protein, a three-dimensional structure of CDK4 was calculated (Figure 5B). It resembles the structure of cdk2, which was determined from crystallized protein by X-ray diffraction [43]. As cdk2, the predicted structure is bi-lobated, containing a β-sheet-rich N-terminal and a alpha-helix-rich C-terminal region. This structure reveals that the N- and C-terminus of CDK4 are neighbouring. Thus, it is possible that both together provide a non-continuous binding domain for Tax. The N-terminus contains the PSTAIRE helix of CDK4, which is part of the CDK's cyclin D2 binding domain. Its rotation during the activation of CDK4 is required to unblock the catalytic cleft of the kinase [44]. Binding of Tax to this region may influence its spacial arrangement. Thus, Tax in cooperation with cyclin D2 could support formation of the active conformation and stimulate CDK4 activity by influencing the PSTAIRE helix.

## Conclusion

The 40 N-terminal amino acids of Tax are sufficient to bind cyclin D2 and CDK4. Within CDK4 a N- and a C-terminal domain are relevant for Tax binding. These domains are neighbouring in the predicted three dimensional protein structure. Taken together, these findings suggest that Tax stimulates G1- to S-phase transition by supporting the association of CDK4 and cyclin D2. Furthermore, they support the conclusion that CDK4 activity is stimulated through conformational changes of the enzyme directly mediated by Tax.

## Methods

### Generation of CDK4 deletion mutants

All CDK4 deletion mutants were generated via PCR [45]. In order to introduce the internal deletions, 16 different primers were used, two outside 28-mer oligonucleotides spanning the 5' and 3' ends of the CDK4 open reading frame (CDK4S and CDK4AS) and 14 chimeric oligonucleotides designed to carry the 5' and 3' sequences flanking the deleted regions. After three rounds of PCR with *Pwo *polymerase (Roche, Mannheim, Germany), the deleted clones CDK4_dH30-V72_, CDK4_dV70-L100_, CDK4_dR101-L120_, CDK4_dM121-S150_, CDK4_dS150-R181_, CDK4_dA182-K211_, CDK4_dK211-D241_, CDK4_dV242-M275 _were created. To engineer the N- terminal CDK4_dM1-F31 _and C-terminal CDK4_dL272-E303 _deletion clones, one round of PCR was performed by using an internal 5' primer or 3' primer in combination with the corresponding outside primer. To engineer the CDK4 full length construct one round of PCR was performed with the outside primers. The resulting PCR products were digested with *Bam*HI and *Hind*III and ligated via these sites into the pcDNA3.1(-)/Myc-His A expression vector (Invitrogen, Karlsruhe, Germany). The resulting clones were verified by nucleotide sequencing.

### Coimmunoprecipitation

Human 293T cells were kept and transfected for coimmunoprecipitations as described [36]. Briefly, cells were lysed in buffer containing 50 mM Tris, 150 mM NaCl, 0.2% Tween 20, 1 mM dithiothreitol, 1 mM phenylmethylsulfonyl fluoride and 10 μg/ml aprotinin. To immunoprecipitate Tax and associated proteins cleared protein supernatant (0.7 to 1 mg whole protein) were incubated for 1 h at 4°C with 1 μg of monoclonal Tax antibody and the immune complexes were collected by protein A-Sepharose CL4B (Pharmacia) beads (1 h at 4°C). Beads with the precipitated proteins were washed three times with lysis buffer. An aliquot of protein supernatant was taken as lysate control (40 μg whole protein). Immunoprecipitates and lysate controls were separated on gels and electro-blotted. Subsequently, membranes were incubated with 5% nonfat dry milk to block unspecific binding before reacting them with a 1: 200 dilution of monoclonal Tax antibody for 1 h at room temperature. Membranes were washed and incubated with a 1:2.500 dilution of an anti-mouse immunoglobulin G-horse-radish peroxidase conjugate (Amersham, Freiburg, Germany). Bound antibodies were visualized with an enhanced chemiluminescence detection system (Amersham) and CCD-camera. The luminescence of specific bands was quantitated from the digitalized image by using the program AIDA (raytest Isotopenmeßgeräte GmbH, Straubenhardt, Germany).

### *In vitro *binding and pull down assays

^35^S-methionine labeled CDK4 and mutants were produced *in vitro *with a rabbit reticolocyte-based *in vitro *transcription/translation system (Promega, Mannheim, Germany). To prevent the expression of the myc/his-tag, the inset plasmids were digested with *HindIII *prior to translation. Tax was produced in *E.coli *and coupled to S-protein-agarose as previousely described [36]. For a binding assay 5–10 μl of the *in vitro*-translated protein was diluted in 500 μl of RIPA buffer (10 mM Tris [pH 7.4], 150 mM NaCl, 2 mM EDTA, 1 % Nonidet P-40, 0.5 % desoxycholat, 0.1 % sodium dodecyl sulfate). An aliquot of 10 μl was taken as an inset control. The S-protein-agarose-bound Tax protein (15 μl) was incubated with the radioactive proteins for 1 h at 4°C, washed with RIPA-buffer and recovered by boiling the beads in loading buffer. Proteins were sized on an SDS-12% polyacrylamide gel, quantitated and visualized by a phosphorimager.

Tax_M1-R40 _was generated via PCR, using the primers Tax_M1-R40 _-pet-S and Tax_M1-R40 _-pet-AS and plasmid pcTax [46] as template. Resulting PCR products and the pet 29b + vector (Novagen, Bad Soden, Germany) were digested with *Bam*HI and *Hind*III and ligated. Resulting clones were verified via sequencing. Cyclin D2 and cyclin E were transfected in 293T cells and lysates were prepared as previously described [36]. A lysate control was performed with 40 μg whole protein. Lysates containing 0.5 – 1 mg whole protein were incubated with *E.coli*-produced Tax_wt _or Tax_M1-R40_, coupled to Ni-NTA agarose for 1 h, washed with lysis buffer and recovered by boiling the beads in loading buffer. Proteins were sized on a 12%-SDS-PAA gel, transferred onto a nitrocellulose transfer membrane and stained with specific antibodies.

### Mammalian two-hybrid assay

All constructs for mammalian two-hybrid assay were generated via PCR. The Tax_M1-R40 _construct PCR was performed with the primer Tax_M1-R40 _-M2H-S and Tax_M1-R40 _-M2H-AS using the plasmid pcTax as a template. For the CDK4 constructs CDK4_dV70-L100 _the *pcDNA3.1(-)/Myc-His A *construct was used as a template. The resulting PCR products were digested with *KpnI *and *XbaI*. For the other CDK4 constructs CDK4_M1-V71, _CDK4_L100-T149, _CDK4_S150-K211 _and CDK4_V242-E303 _the CDK4 full length *pcDNA3.1(-)/Myc-His A *construct was used as template. The resulting PCR products were digested with *BamHI *and *XbaI*. The digested products were ligated into the vectors pBind and pAct (CheckMate Mammalian two-hybrid system, Promega). The vector pG5*luc *contains the reporter gene (*Firefly *luciferase). Human 293 cells were transfected with the plasmids using Lipofectamine reagents (Invitrogen). The luciferase-assay was performed with the Dual-Luciferase reporter assay (Promega) using a microplate luminometer.

### Oligonucleotides

Designation for primers correspond to the plasmid names. The oligonucleotides sequences were as follows:

CDK4S, 5'-ATTTACGGATCCACCATGGCTACCTCTC-3' (outer primer);

CDK4AS, 5'-ATCCCCAAGCTTCTCCGGATTACCTTCA-3' (outer primer);

CDK4_dM1-F31_S, 5'-ATTTACGGATCCATGGTGGCCCTCAAGA-3';

CDK4_dH30-V72_S, 5'-CACAGTGGCCACTTTGTCCGGCTGSTGGAC-3';

CDK4_dH30-V72_AS, 5'-GTCCATCAGCCGGACAAAGTGGCCACTGTG-3';

CDK4_dV70-L100_S, 5'-GCTTTTGAGCATCCCAATAGGACATATCTGGACAAG-3';

CDK4_dV70-L100_AS, 5'-CTTGTCCAGATATGTCCTATT GGATGCTCAAAAGC-3';

CDK4_dM121-S150_S, 5'-GAAACGATCAAGGATCTGGGTGGAACAGTCAAGCTG-3';

CDK4_dM121-S150_AS, 5'-CAGCTTGACTGTTCCACCCAGATCCTTGATGGTTTC-3';

CDK4_dS150-R181_S, 5'-AACATTCTGGTGACAAGTGTTACACTCTGGTACCGA-3';

CDK4_dS150-R181_AS, 5'-TCGGTACCAGAGTGTAACACTTGTCACCAGAATGTT-3';

CDK4_dA182-K211_S, 5'-GCTCCCGAAGTTCTTCTGCCTCTCTTCTGTGGAAAC-3';

CDK4_dA182-K211_AS, 5'-GTTTCCACAGAAGAGAGGCAGAAGAACTTCGGGAGC-3';

CDK4_dK211-D241_S, 5'-GCAGAGATGTTTCGTCGAGATGTATCCCTGCCCCGT-3';

CDK4_dK211-D241_AS, 5'-ACGGGGCAGGGATACATCTCGACGAAACAGCTCTGC-3';

CDK4_dV242-M275_S, 5'-GATGACTGGCCTCGAGATCTGACTTTTAACCCACAC-3';

CDK4_dV242-M275_AS, 5'-GTGGGTGTTAAAAGTCAGATCTCGAGGCCAGTCATC-3';

CDK4_dL272-E303_AS, 5'-ATTTAGAAGCTTCAGCAGCTGTGCTCCC-3';

Tax_M1-R40 _-M2H-S, 5'-TCATCTAGAATGGCCCATTTCCCAGGGTT-3'(outer primer);

Tax_M1-R40 _-M2H-AS, 5'-ATTGGTACCTAGGCGGGCCGAACATAGTC-3'(outer primer);

CDK4-M2H-S, 5'-CCTTGGATCCTAATGGCTACCTCTC-3'(outer primer);

CDK4-M2H-AS, 5'-GCATTCTAGACGCCTCCGGATTACCTT-3'(outer primer);

CDK4_M1-V71_-AS, 5'-GCATTCTAGACGCAACATTGGGATGCTCAAA-3';

CDK4_L100-T149_-S: 5'-CCTTGGATCCTACTAAGGACATATCTGGAC-3';

CDK4_L100-T149_-AS: 5'-GCATTCTAGACGCTGTCACCAGAATGTTCTC-3';

CDK4_S150-K211 _-S: 5'-CCTTGGATCCTAAGTGGTGGAACAGTCAAG-3';

CDK4_S150-K211 _-AS: 5'-GCATTCTAGACGCCTTTCGACGAAACATCTC-3';

CDK4_V242-E303_-S: 5'-CCTTGGATCCTAGATGTATCCCTGCCCCGT-3';

Tax_M1-R40 _-pet-S: 5'-GATCGGATCCGATGGCCCATTTCCCAGGGTT-';

Tax_M1-R40 _-pet-AS: 5'-CTAATTAAGCTTTAGGCGGGCCGAACATAGTCCCCCAGAGATG-3',

## Competing interests

The author(s) declare, that they have no competing interests.

## Authors' contributions

KF performed most of the experiments. BM did experiments shown in Figure 1. Both KF and RG participated in experimental design, data interpretation and writing of manuscript. All authors have critically read the manuscript and approved the final version to be published.
